# Anisotropic Thermally Conductive Polyurethane Composites Based on Tannic Acid-Modified Silicon Carbide/Woven Fiber Skeletons

**DOI:** 10.3390/polym18111414

**Published:** 2026-06-05

**Authors:** Qingqing Yang, Lili Wu

**Affiliations:** School of Materials Science and Engineering, Wuhan University of Technology, Wuhan 430070, China

**Keywords:** thermal conductivity, polyurethane, silicon carbide, tannic acid

## Abstract

With the miniaturization of electronic devices, the demand for high-efficiency thermal management materials has become increasingly urgent. Although traditional high-filler random blending composites can enhance thermal conductivity, they often do so at the expense of mechanical properties and lightweight advantages. Therefore, constructing oriented thermal conduction networks at low filler loadings has become a core challenge in current research. This study proposes an interface engineering strategy based on a tannic acid (TA) molecular bridging layer to modify silicon carbide (SiC). By leveraging the self-polymerization and strong adhesion properties of TA, a dense fish scale SiC coating was formed on the surface of highly oriented woven cellulose acetate (WF) through a simple impregnation process. After compositing with a polyurethane (PU) matrix, the obtained WF/TA/SiC/PU exhibits anisotropic thermal conductivity. It has an axial thermal conductivity of 0.44 W/mK, an increase of 411% over PU, and the decomposition temperature has increased by 18.2 °C. Additionally, the composite axial thermal response rate significantly outperforms both the radial direction and PU. This research demonstrates a new approach for achieving high-efficiency thermal management at low filler loadings, providing a scalable pathway for the development of sustainable, lightweight, and high-performance anisotropic heat dissipation devices.

## 1. Introduction

With the rapid integration and miniaturization of electronic devices, effective heat dissipation has become a critical factor determining their operational stability and lifespan [1,2,3]. Polyurethane (PU) is widely utilized in electronic packaging and cable insulation due to its excellent resistance to acids and alkalis, superior elasticity, and electrical insulation [4,5,6,7,8,9]. However, the inherently low thermal conductivity of the PU matrix significantly restricts its application in high-power density electronics.

Conventional strategies to enhance thermal conductivity involve incorporating high thermal conductivity fillers into the PU matrix [10,11,12,13]. Wolfsgruber et al. investigated the effects of various inorganic fillers (aluminum oxide, zinc oxide, and boron nitride) on the properties of thermoplastic composites [14]. Results indicate that, besides the volume fraction, the particle shape in combination with the intrinsic thermal conductivity of the filler has the greatest influence on the thermal conductivity of the composite. However, random blending of a single or multiple fillers often fails to create effective heat transfer pathways at low loading levels, while high filler loading inevitably compromises the mechanical properties, processability, and weight-saving advantages of the composite material [15,16,17]. Therefore, designing and constructing efficient, continuous thermal conduction paths at low filler loadings is a pivotal challenge in material science.

To resolve this conflict, constructing oriented structures as templates has become an effective approach. Wang et al. utilized PU foam with a robust skeletal structure as a three-dimensional network template. Through layer-by-layer self-assembly, they coated the surface with hydroxylated boron nitride (BNNS-OH) and combined this with microwave-assisted curing technology, successfully enhancing the anisotropic thermal conductivity of polyvinyl alcohol composites significantly, even at low loading levels. When the BNNS-OH coating is 10 wt%, the optimal thermal conductivity is achieved [18]. Although three-dimensional foam templates provide multiple heat transfer pathways, one-dimensional or two-dimensional height-oriented fiber networks, such as woven mesh fibers (WF), offer greater potential for directed heat transfer due to their more precise alignment and structural continuity [19,20,21].

In addition, the interfacial adhesion between the filler and the matrix is crucial for reducing interfacial thermal resistance. Inspired by the adhesion mechanism of mussels, polydopamine modification technology has been widely adopted [1,22,23,24,25,26]. Liu et al. used thermoplastic polyurethane (TPU) as the matrix, modified boron nitride (BN) with polydopamine and complexed it with Fe_3_O_4_, and then performed a secondary modification with polydopamine, successfully preparing TPU/m-BN composites with high thermal conductivity and good mechanical properties [27]. However, the high cost of dopamine limits its widespread application. It is worth noting that plant-derived (TA), which contains pyrogallol and catechol groups similar to dopamine, has emerged as a sustainable and cost-effective alternative. TA can form stable, functional bridging layers through oxidative self-polymerization, providing abundant phenolic hydroxyl groups for filler functionalization and enabling their oriented anchoring to the template via hydrogen bonding and van der Waals forces [28,29,30,31,32,33].

Based on these synergistic strategies of oriented templates and biomimetic interfaces, this paper proposes a more cost-effective strategy. As a natural alternative to dopamine, the plant polyphenol TA possesses abundant terminal phenolic hydroxyl groups that can undergo oxidative self-polymerization to form stable functional bridging layers on various substrate surfaces. Silicon carbide (SiC) possesses excellent intrinsic thermal conductivity, which far exceeds that of traditional oxides such as Al_2_O_3_. Simultaneously, due to slight surface oxidation, the SiC surface is naturally rich in hydroxyl (-OH) groups, which are highly compatible with the phenolic hydroxyl groups abundant in TA, thereby ensuring strong hydrogen bonding and a robust interfacial bond. Furthermore, compared to humidity-sensitive aluminum nitride or costly boron nitride, SiC offers superior cost-effectiveness and achieves a good balance between thermal conductivity and electrical insulation under low-load conditions. Therefore, this study utilizes the highly oriented network of WF materials as a scaffold and employs the bridging effect of TA to efficiently anchor SiC particles onto the fiber surface. Compared to previous studies, the significance of this work lies in two aspects: first, by leveraging the orientation properties of WF to induce the formation of continuous phonon transport channels, it addresses the performance degradation issues associated with high filler content traditional random blending systems. Second, by replacing expensive dopamine with TA, it achieves a scaly close-packing coating at the interface. This approach not only enables efficient thermal management at low filler loading but also endows the composite with significant anisotropy, providing a new approach for developing sustainable, lightweight heat dissipation materials for high-performance electronic devices.

## 2. Experiment

### 2.1. Materials

Casting polyurethane (PU, type AB) was supplied by Jining Baiyi Chemical Co., Ltd., Jining, China. Woven reticulated acetate fiber was provided by China Tobacco Hubei Industrial Co., Ltd., Wuhan, China. Tannic acid (ACS grade) was purchased from Aladdin Biochemical Technology Co., Ltd., Shanghai, China. Tris (hydroxymethyl) aminomethane (Tris, AR grade) and silicon carbide (average particle size 2 μm) were obtained from Aladdin Reagent Co., Ltd., Shanghai, China. All chemicals were used as received without further purification.

### 2.2. Preparation of Tannic Acid-Modified Silicon Carbide/Woven Fiber Skeletons (WF/TA/SiC)

**Figure 1:**
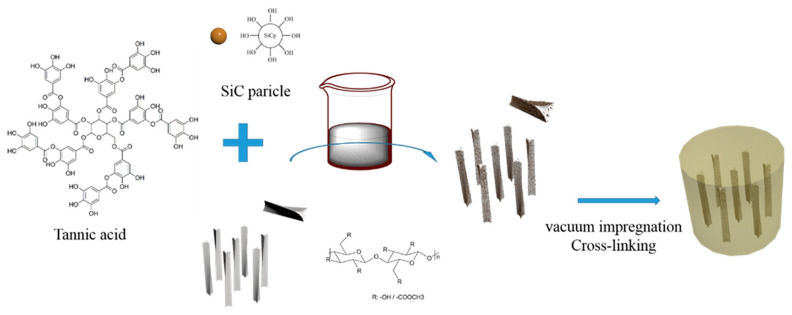
Schematic diagram of the manufacturing process of WF/TA/SIC polyurethane resin composites.

Figure 1 shows the fabrication flowchart of the composite material. First, 2 g of SiC micropowder was added to 300 mL of deionized water and dispersed via ultrasonication for 6 h. Subsequently, 1.5 g of tannic acid was added, and the pH of the mixture was adjusted to 8.5 using tris buffer. The WF mesh was suspended horizontally and immersed in the solution. After gentle stirring for 2, 5, and 10 h, the resulting 3D cellulose skeletons were removed and dried in a blast drying oven at 30 °C for 1 h for subsequent use.

### 2.3. Preparation of Polyurethane Composites Based on Tannic Acid-Modified Silicon Carbide/Woven Fiber Skeletons (WF/TA/SiC/PU)

Initially, 20 g of polyurethane part A and 22 g of part B were weighed and heated in a vacuum oven at 60 °C for 15 min to reduce the viscosity. After thorough stirring, the PU melt was transferred into a clean, dry mold. The aforementioned 3D skeletons were then immersed in the melt, and the assembly was placed in a vacuum drying oven. Degassing was performed under a vacuum of −75.01 torr (−0.1 MPa) for 12 h. Finally, the vacuum oven was set to 60 °C for 3 h for initial curing, followed by a secondary curing stage at 110 °C for 3 h.

### 2.4. Characterization

The chemical structures of modified SiC were characterized by Fourier transform infrared (ATR-FTIR, iN10-iS50, Waltham, MA, USA) spectroscopy, and the surface morphology and elemental distribution of the samples were characterized via scanning electron microscopy coupled with energy dispersive spectroscopy (SEM-EDS, JSM-7500F, Tokyo, Japan). The thermal conductivity was measured using a thermal constant analyzer (TPS 2500S, Gothenburg, Sweden) via the transient planar heat source method, with circular specimens (3.5 cm in diameter and 5 mm thick) polished by abrasive paper to ensure surface flatness. Thermal management performance, including heating and cooling processes, was evaluated using a FLUKE Ti400, Everett, WA, USA infrared thermal imager. For the heating test, the polished circular samples were placed on a 100 °C constant temperature hot plate. For the cooling test, samples were preheated at 100 °C in a vacuum oven for 3 h and then transferred to an insulated foam base. Infrared images were automatically captured every 5 s over a total duration of 600 s. Additionally, the thermal stability of the samples was tested using a comprehensive thermal analyzer (STA449F3, Selb, Germany) under a N_2_ atmosphere, with a heating range from 20 °C to 800 °C at a rate of 10 °C/min.

## 3. Results and Discussion

### 3.1. Composition Analysis of WF/TA/SiC Fiber Skeletons

**Figure 2:**
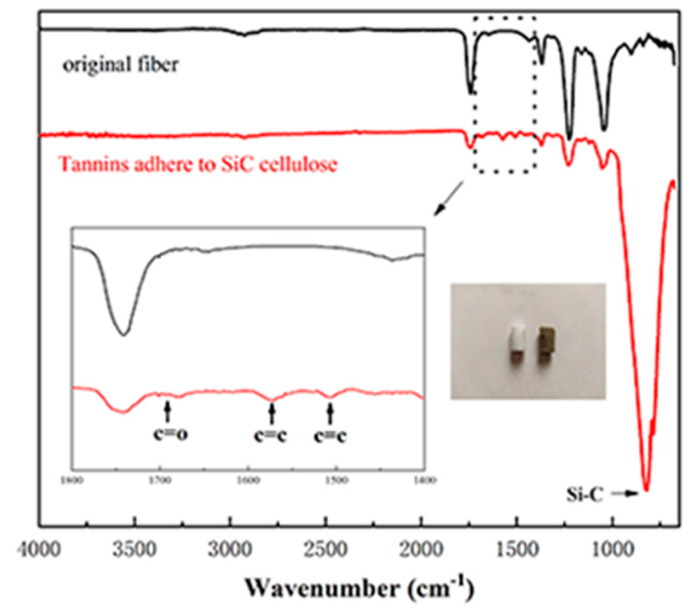
FTIR spectra of original WF skeleton and WF/TA/SiC skeleton.

To verify the promotion effect of the TA oxidative self-polymerization process on the enrichment of SiC on the WF surface, FTIR was employed to analyze the chemical composition of the samples. As shown in Figure 2, the original WF skeleton exhibited a white 3D reticulated structure. After the TA oxidative self-polymerization treatment, the color of the skeleton deepened significantly, changing from white to dark gray, which initially indicated that the SiC particles had been successfully loaded onto the surface of the WF skeleton. The FTIR results showed that the original WF skeleton displayed typical characteristic absorption peaks of cellulose acetate. After modification, a distinct Si-C bond stretching vibration peak appeared at 823 cm^−1^ in the sample. Meanwhile, vibration peaks assigned to the benzene ring skeleton were observed at 1506 cm^−1^ and 1571 cm^−1^, while a stretching vibration peak of the carbonyl group (C=O) in a six-membered ring appeared at 1681 cm^−1^. Since neither SiC nor the WF matrix itself contains a benzene ring structure, the emergence of these characteristic peaks fully demonstrates that tannic acid underwent self-polymerization during the oxidation process and acted as a bridging layer to effectively fix the SiC onto the fiber surface. In summary, the FTIR results confirmed that the oxidative self-polymerization of TA successfully achieved the enrichment and stable loading of SiC on the WF skeleton.

### 3.2. Surface Morphology of WF/TA/SiC Composite Skeletons

**Figure 3:**
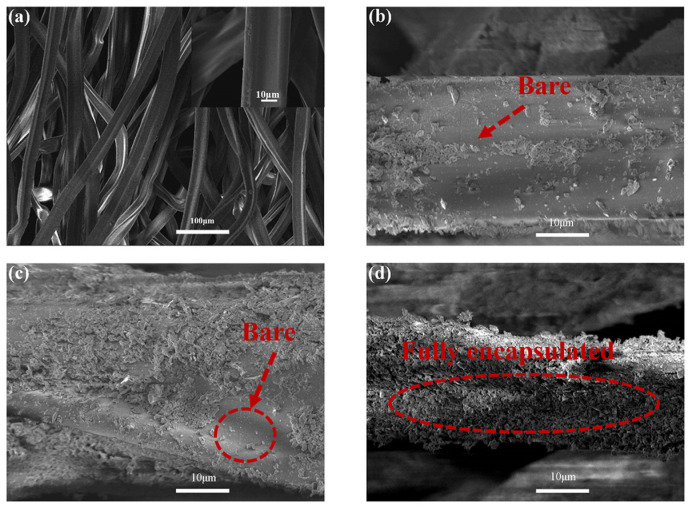
SEM images of (**a**) the original WFs and modified (**b**) 2 h, (**c**) 5 h, and (**d**) 10 h fibers.

The morphology of the pristine WFs and the SiC enrichment on the fiber surface, assisted by the oxidative self-polymerization of TA, were characterized via SEM. As shown in Figure 3a, the pristine WFs exhibit a three-dimensional interlaced structure with a certain degree of orientation in their overall arrangement, where the fibers are distributed roughly in the same direction. This nearly parallel structure provides the structural foundation for the composite to achieve anisotropic properties. The magnified view shows that the fiber surface is smooth and flat, with a single fiber diameter of approximately 30 μm.

Figure 3b–d display the morphologies of the WF/TA/SiC skeletons prepared at different reaction times. From Figure 3b, it can be seen that the SiC particles are scattered on the fiber surface with only local contact, and a continuous network structure has not yet been formed. With the extension of the reaction time Figure 3c, the SiC particles gradually enrich and interconnect on the fiber surface, initially forming a continuous thermal conduction path, although some uncovered areas still exist. Further extending the reaction time (Figure 3d), the fiber surface is completely encapsulated by densely packed SiC particles, exhibiting a distinct rough structure, and the original surface features of the fibers have basically disappeared. The above results indicate that the TA oxidative self-polymerization can effectively regulate the deposition and connection state of SiC on the fiber surface. As the SiC particles transition from a dispersed state to a continuous network, a complete heat conduction path is gradually constructed, which is conducive to improving the thermal conductivity of the composite. Furthermore, the WF/TA/SiC skeleton maintains the original oriented structure of the fibers, exhibiting typical high aspect ratio anisotropic characteristics, providing a structural basis for the material to achieve direction-dependent thermal conduction.

### 3.3. Morphological Analysis of WF/TA/SiC/PU Composites

**Figure 4:**
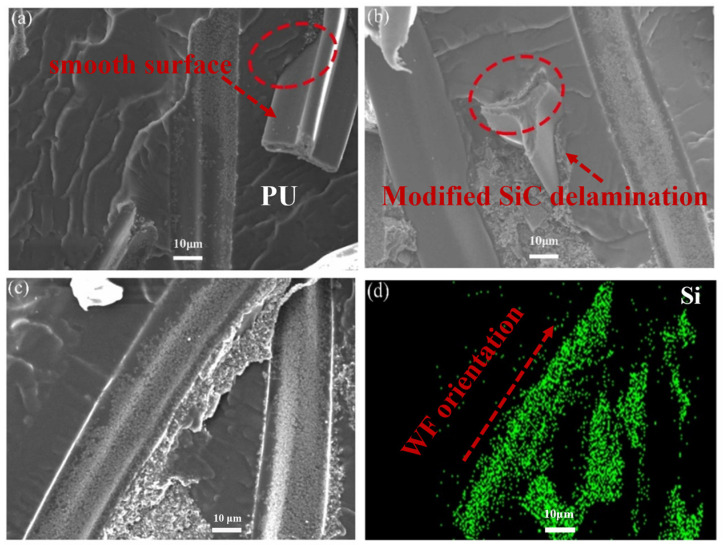
SEM images of the cross-sectional morphology of the modified WF fiber skeleton and PU after compounding: (**a**) 2 h, (**b**) 5 h, (**c**) 10 h. (**d**) Map of Si distribution in the cross-section of the composite material after 10 h of modification.

The fracture surface morphology of the modified WF/TA/SiC skeletons after compounding with PU resin is shown in Figure 4. Figure 4a indicates that SiC particles are locally enriched within the PU matrix, forming limited thermal conductive connections. Meanwhile, some fiber surfaces remain smooth, with only localized residual SiC particles observed. This suggests that SiC particles tend to remain within the resin phase during the fracture process, reflecting a stronger interfacial interaction with the PU matrix.

As the modification time increases, a continuous particle layer is formed on the fiber surfaces and their adjacent regions. However, localized debonding still occurs, indicating a certain degree of heterogeneity in interfacial bonding (Figure 4b). Additionally, while transverse SiC accumulation exists between fibers, a fully interconnected lateral network structure has not yet been established. Consequently, the composite primarily constructs continuous thermal conduction pathways along the axial direction of the fibers, whereas the heat transfer paths in the radial direction remain restricted. This leads to a significant structural anisotropy in thermal conduction (Figure 4c). The distribution of the Si element (Figure 4d) further confirms these orientation-dependent filler distribution characteristics. Overall, the WF/TA/SiC skeleton maintains its structural orientation integrity during the compounding process, establishing a thermal conductive network with preferential axial connectivity. This anisotropic filler architecture serves as the core structural factor for enhancing the thermal conductivity and achieving direction-dependent properties in the composite material.

### 3.4. Thermal Conductivity of WF/TA/SIC/PU Composites

**Figure 5:**
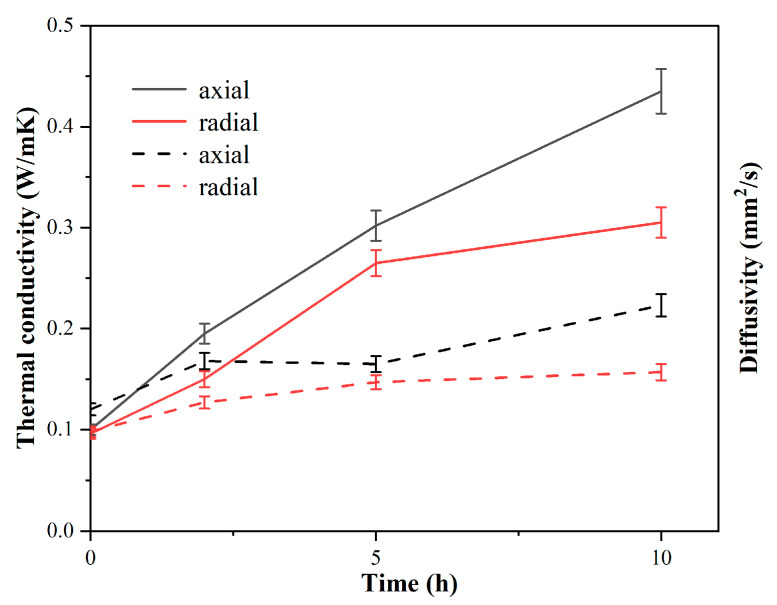
The thermal diffusivity (dashed line) and thermal conductivity (solid line) curves of the WF/TA/SIC/PU composite in the axial direction and radial direction after 10 h of tannin reaction.

Figure 5 illustrates the evolution of thermal conductivity for the WF/TA/SiC/PU composites in both axial and radial directions. The results indicate that the thermal conductivity of the composites improves progressively as the SiC loading on the fiber surfaces increases with extended modification time. After 2 h of modification, the axial thermal conductivity of the composite reaches 0.20 W/mK, which is approximately 100% higher than that of pure PU, whereas the radial thermal conductivity is 0.15 W/mK. This anisotropy in thermal conduction is attributed to the construction of relatively continuous heat transfer pathways by SiC particles along the fiber axis.

In contrast, although the lateral contact between particles can establish a limited number of conduction paths, the presence of substantial PU matrix gaps between fibers hinders the formation of a continuous network as pervasive as that in the axial direction. When the modification time is extended to 10 h, the SiC particles form a more complete coating structure on the fiber surfaces, leading to a significant increase in the axial thermal conductivity to 0.44 W/mK (a 411% improvement over pure PU), while the radial thermal conductivity increases to 0.31 W/mK. This result is consistent with the SEM morphological observations, demonstrating that the gradual formation of a continuous axial heat conduction network with increasing SiC loading significantly enhances the thermal transport performance. Conversely, although partial conduction pathways are also formed in the radial direction, the magnitude of the improvement remains lower than that in the axial direction due to the insufficient continuity of the network.

### 3.5. Thermal Management Performance of WF/TA/SiC/PU Composites

**Figure 6:**
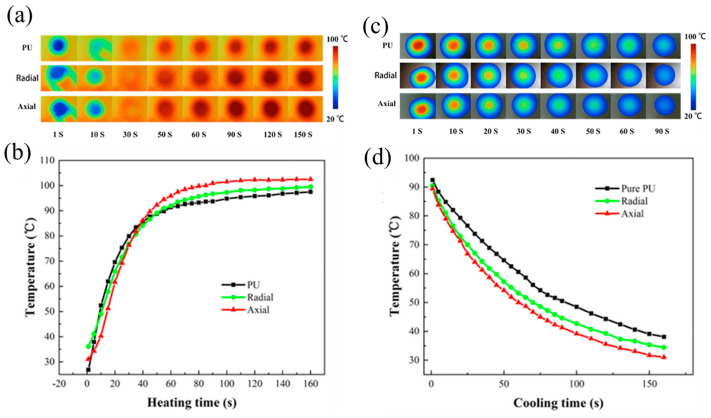
Axial and radial thermal management performance of pure PU and WF/TA/SIC/PU composites modified for 10 h: Infrared thermal images during (**a**) heating and (**c**) cooling, and (**b**) heating and (**d**) cooling curves.

The evolution of surface temperature over time during the heating and cooling processes was recorded using a Ti400 infrared thermal imager to intuitively characterize the anisotropic thermal conduction and dissipation performance of the materials. The pure PU and WF/TA/SiC/PU composite modified for 10 h were placed on a constant-temperature heating stage for thermal testing. As shown in Figure 6a, as the surface temperature increases, the color of the samples in the IR images gradually shifts from deep blue, representing low temperatures, to red, representing high temperatures. After heating for 80 s, the surface temperatures of the WF/TA/SiC/PU composite in the axial and radial directions reach 99.8 °C and 95.7 °C, respectively, while that of pure PU is only 93.2 °C. The heating rate of the WF/TA/SiC/PU composite in the axial direction is significantly faster than that in the radial direction, which is highly consistent with the anisotropy of its thermal conductivity.

As illustrated in Figure 6b, the axial sample not only exhibits the fastest heating rate but also is the first to reach the highest stable temperature; the stable temperature of the radial sample follows, while pure PU shows the lowest. Furthermore, the heat dissipation performance was further evaluated by recording the natural cooling process of the samples from a heated state (Figure 6c). At 150 s of cooling, the axial temperature of the WF/TA/SiC/PU composite decreases to 31 °C, whereas the radial temperature and the temperature of pure PU are 34.4 °C and 38.1 °C, respectively. These results demonstrate that the composite exhibits the highest heating and cooling rates in the axial direction. In summary, the thermal diffusion capacity of the WF/TA/SiC/PU composite in both the axial and radial directions is superior to that of pure PU, with the axial direction exhibiting a more prominent anisotropic heat dissipation advantage. This confirms the high efficiency of the continuous SiC thermal conduction network constructed along the fiber axis for thermal management applications.

### 3.6. Thermal Stability of WF/TA/SIC/PU Composites

**Figure 7:**
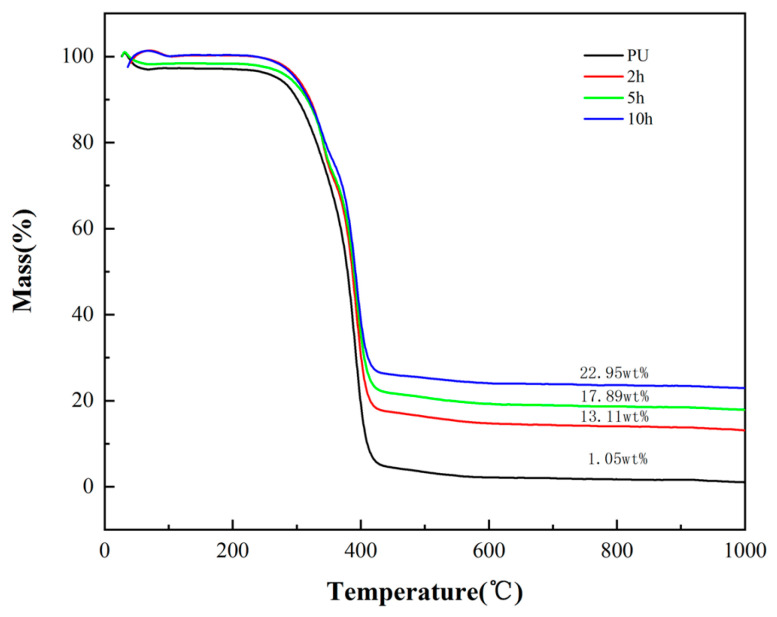
TGA curves of PU and WF/TA/SIC/PU (2, 5, 10 h) composites.

Thermogravimetric analysis (TGA) was employed to evaluate the thermal decomposition behavior and thermal stability of the pure PU and WF/TA/SiC/PU composite. As shown in Figure 7, all samples begin to undergo significant decomposition at approximately 230 °C, and the decomposition rate tends to stabilize near 440 °C. The results indicate that the introduction of SiC fillers improves the initial decomposition temperature of the composites.

Taking the temperature at 10 wt% mass loss (T_10_) as an example, the T_10_ of pure PU is 297.43 °C. Meanwhile, the T_10_ values for the composites modified with TA for 2 h, 5 h, and 10 h increase to 317.76 °C, 312.75 °C, and 315.63 °C, respectively. The enhancement in thermal stability can be attributed to the SiC particles acting as high thermal conductivity components that serve as thermal dispersion centers during the heating process, thereby delaying the thermal degradation of the polymer matrix. Consequently, the decomposition temperature required for the composites to reach the same mass loss rate is higher. When the pyrolysis process reaches a stable state, the residual mass percentages of the composites modified for 2 h, 5 h, and 10 h are 13.11 wt%, 17.89 wt%, and 22.95 wt%, respectively. Considering that WFs undergo complete decomposition at the test temperature, the aforementioned residual mass can be regarded as the actual loading of SiC particles in the composites. The experimental results confirm that at a maximum SiC loading of 22.95 wt%, the composite achieves a high thermal conductivity of 0.44 W/mK.

## 4. Conclusions

This paper proposes a strategy for modifying SiC using TA as a functional bridging layer, achieving high efficiency enrichment and oriented alignment of SiC particles on the surface of WF through a simple impregnation. By compounding with PU resin, WF/TA/SiC/PU composites with anisotropic thermally conductive networks were successfully constructed. Research indicates that the TA modification process effectively regulates the distribution state of SiC particles, forming a fully encapsulated and densely packed fish-scale-like SiC coating on the WF surface. This unique structure maintains excellent structural integrity of the orientation during the compounding process with PU resin, establishing robust and continuous phonon transmission channels along the fiber axial direction. The obtained WF/TA/SiC/PU composite exhibits significant thermal anisotropy and enhanced thermal stability. After 10 h of impregnation, the composite achieved an outstanding axial thermal conductivity of 0.44 W/mK (a 411% increase over pure PU), while its thermal stability is also improved, with the T_10_ decomposition temperature increasing by 17.93 °C. Infrared thermography analysis further confirms the anisotropic advantage. The composite exhibits a faster thermal response rate in the axial direction compared to the radial direction and pure PU. This performance is attributed to the synergistic effect between the oriented fiber template and SiC acting as thermal dispersion centers, which accelerates heat diffusion while effectively retarding the thermal degradation of the matrix. Crucially, this work demonstrates a novel approach to achieving high efficiency thermal management at low filler loadings, reaching performance levels comparable to traditional random blending systems.

## Data Availability

The raw data supporting the conclusions of this article will be made available by the authors on request.
